# Laparoscopic procedures impact on mast cell mediators, extracellular matrix and adhesion scoring system in rats

**DOI:** 10.1016/j.amsu.2020.08.043

**Published:** 2020-09-02

**Authors:** Hery Poerwosusanta, Gunadi, Ika Kustiyah Oktaviyanti, Nia Kania, Zairin Noor

**Affiliations:** a Department of Surgery, Faculty of Medicine, Universitas Lambung Mangkurat, Banjarmasin, South Kalimantan, Indonesia; b Pediatric Surgery Division, Department of Surgery, Faculty of Medicine, Public Health and Nursing, Universitas Gajah Mada /Dr. Sardjito Hospital, Yogyakarta, Indonesia; c Department of Anatomical Pathology, Faculty of Medicine, Universitas Lambung Mangkurat, Banjarmasin, South Kalimantan, Indonesia

**Keywords:** Laparoscopy, Mast cell mediators, Histamine, Protease, Extracellular matrix thickness, Intra-abdominal adhesion, **ATP**, Adenosine triphosphate, **CRAC**, Calcium release-activated channels, **CO**_**2**_, Carbon dioxide, **DAMPs**, Damage Associated Molecular Patterns, **DNA**, Deoxyribonucleic acid, **ELISA**, Enzyme-linked-immunosorbent-assay, **ECM**, Extracellular matrix, **GPCR**, G Protein-Coupled Receptors, **pro-MMP9**, pro Matrix metallopeptidase 9, **PAR-2**, protease-activated receptor 2, **ROS**, Reactive Oxygen Species, **TGF-β**, Transforming growth factor-beta, **TRPC**, Transient receptor potential canonical, **TRPV4**, Transient receptor potential vanilloid 4, **tPA**, tissue plasminogen activator, **uPA**, urokinase plasminogen activator, **VDAC**, Voltage-dependent anion channel

## Abstract

**Background:**

Laparoscopic procedures under certain pressure have the potential to cause intra-abdominal adhesions. However, the pathomechanism of this disorder is unknown. Release of mast cell mediators due to mast cell degranulation is thought to be the cause.

**Materials and methods:**

Thirty male Sprague-Dawley rats were grouped into five groups (n = 6 per group): one control group and four intervention groups to which 60 min insufflation was performed using carbon dioxide at 5, 8, 10 and 12 mmHg. Seven days after laparoscopy, we euthanized and evaluated the levels of histamine, tryptase, and chymase of peritoneal fluid, the thickness of ECM of peritoneal tissue, and intraabdominal adhesion scoring system.

**Results:**

Histamine and tryptase levels in peritoneal fluid were significantly higher at the 10- and 12 mm Hg intervention compared to control (histamine: 0.50 ± 0.35 vs. 0.41 ± 0.41 vs. 0.04 ± 0.02 ng/mL, respectively; and tryptase: 0.69 ± 0.11 vs. 0.65 ± 0.05 vs. 0.48 ± 0.02 ng/ml respectively). The ECM was significantly thicker in the intervention groups at 10- and 12-mm Hg compared to control (71.3 [66.7–85.2] vs. 48.4 [34.5–50.3] vs. 10.25 [8.7–12.1] μm, respectively). Moreover, the intra-abdominal scoring was also significantly higher in the intervention groups at 10- and 12 mm Hg compared to control (4 [0–4] vs. 4.5 [4–5], vs. 0, respectively).

**Conclusions:**

Laparoscopic procedures increase the release of mast cell mediators in peritoneal fluid, the thickness of ECM and intraabdominal adhesion scoring in rats, implying that it might increase the possibility of intrabdominal adhesion in humans.

## Highlights

• Laparoscopic procedures at specific pressures potentially cause intra-abdominal adhesion, however, its pathomechanism is still challenging to understand.
• Laparoscopic procedures increase the release of mast cell mediators in peritoneal fluid, the thickness of ECM and intraabdominal adhesion scoring in rats.
• Our findings imply that laparoscopic procedures might increase the possibility of intrabdominal adhesion in humans.

## 1 Introduction

Carbon dioxide (CO_2_) insufflation in laparoscopy procedures causes mesothelial morphological changes [1], structure damage [2], and the risk of intra-abdominal adhesion [3]. Tissue damage triggers the inflammatory response, mast cell infiltration, and degranulation that are believed to stimulate adhesion. The study about the effect of mast cell mediators on the intra-abdominal adhesion pathomechanism is still rarely conducted.

Mast cells are specific [4], mature in the tissues, and form 10% of the mesothelium immune cell population [5]. Laparoscopy procedures cause mast cell infiltration and degranulation [6]. The release of histamine, tryptase, and chymase due to mast cell degranulation [7] are presumed to play a role in intra-abdominal adhesion. Our objective was to determine the impact of the laparoscopic procedure on 1. mast cell mediators’ level, including histamine, tryptase, and chymase; 2. the thickness of the extracellular matrix (ECM) of peritoneal tissue; and 3. intraabdominal adhesion scoring system.

## 2 Materials and methods

### 2.1 Animal subjects

This study was conducted according to the 3R5F principles of experimental animal studies [8,9]. Thirty males [10], 200–250 g, and 20–25 weeks old Sprague-Dawley rats (*Rattus norvegicus*) were randomly computerize divided into a control group and four intervention groups. The rats were kept in standard breeding-housing (maintained 20 ± 2 °C temperature, 12 h light/dark cycle), standard food, mineral water, health monitor, and 7 days of acclimation) [11]. The sick and dead rats were excluded from the study and replaced with healthy. The control group (n = 6) did not receive pneumoperitoneum. The intervention groups of P-5 mmHg, P-8 mmHg, P-10 mmHg, and P-12 mmHg (all n = 6) were given 5, 8, 10, and 12 mmHg CO_2_ pneumoperitoneum, respectively [6,12]. Our study strictly followed the ethical and euthanasia guidelines for animal research (http://risetcenterfk.ulm.ac.id/euthanasia/). The Animal Experimentation Ethical Committee, Research Center, Faculty of Medicine, Universitas Lambung Mangkurat, Banjarmasin, Indonesia, had approved our research (No.282/KEPK-FK.UNLAM/EC/VII/2019). The experiments were conducted in the Chemical/Biochemical Laboratory, the Anatomical Pathology Laboratory, Faculty of Medicine, Universitas Lambung Mangkurat, Banjarmasin, Indonesia.

### 2.2 Laparoscopy procedures

According to the previous study [6], 60-min laparoscopy was performed in a sterile area after shaving and povidone-iodine application. Ten mg/kg BW intramuscular injections of ketamine hydrochloride (KTM-10; PT Guardian Pharmatama, No. Reg. DKL0408013443B1) were used for anesthesia. Pneumoperitoneum used standard CO_2_ and CO_2_ automatic-insufflators (Gimmi, Gimmi®GmbH, Germany, 2000).

### 2.3 Sample collection

Decapitation was performed to euthanize the rats on the 7th-day after laparoscopy [13]. The peritoneum was stained with Masson trichrome to evaluate the ECM thickness in 40x magnification [14].

### 2.4 Histamine, tryptase and chymase analysis

The peritoneal fluid, mast cells, histamine and protease levels were measured using a commercial kit, the enzyme-linked-immunosorbent-assay (ELISA). Histamine and protease levels used Cloud-clone Corp. ELISA Kit for Histamine (HA) for pan-species CEA927Ge [15], Tryptase (TPS) for Rat SEB070Ra [16], and Chymase-1 Mast Cell (CMA1) for Rat SEG515Ra [17].

### 2.5 Extracellular matrix thickness and intra-abdominal scoring evaluation

The ECM thickness was measured by using the Masson trichrome stain, based on Skytec TRM-1-IFU's collagen Trichrome Stain (Connective Tissue Stain) [18] collagen deposition and quantified with ImageJ version 1.51j8 RRID: SCR_003070 [19]. Modified intra-abdominal adhesion scoring for laparoscopy [2] was used (Table 1).

**Table 1:** Modified intra-abdominal adhesion after laparoscopic surgery.

Variables	Score
Extension of adhesion	
⁃ No adhesion	0
⁃ <25% of adhesion area	1
⁃ 25–50% of adhesion area	2
⁃ >50% of adhesion area	3
Severity of adhesion	
⁃ No adhesion	0
⁃ Easy separated, blunt and no sharp dissection	1
⁃ Sharp dissection <50%	2
⁃ Sharp dissection >50%	3
Bleeding level during dissection of adhesion	
⁃ No adhesion	0
⁃ No bleeding on dissection	1
⁃ Bleeding stops spontaneously on dissection	2
⁃ Bleeding does not stop spontaneously on dissection	3

### 2.6 Statistical analysis

Our study results were presented as numbers, percentages, mean ± standard deviation (SD), and median (range, minimum-maximum). Data were analyzed for normality (using Kolmogorov–Smirnov, and Shapiro–Wilk tests), homogeneity using Levine's test, and underwent data transformation methods (power > 1, inverse, log 10, and square root). One-way ANOVA and the post-hoc LSD tests were used for normally and homogeneously distributed data. One-way test of Equality of Means and the post-hoc Games-Howell test were used for normally but non-homogeneously distributed data. Kruskal-Wallis and post-hoc Mann-Whitney tests were used for non-normally distributed data. With a confidence interval of 95% (α = 0.05), the analysis used IBM SPSS version 23.0 and Microsoft Excel 2010.

## 3 Results

### 3.1 Mast cell mediators’ level after laparoscopic procedures

**Fig. 1:**
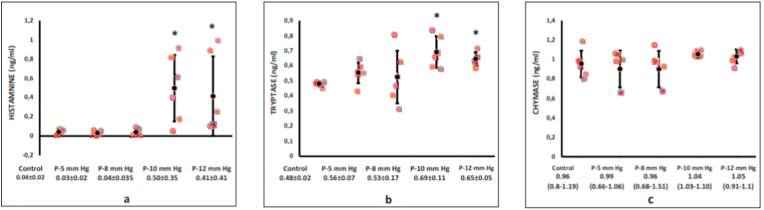
The mast cell mediators' level after laparoscopic surgery. Histamine (a) and tryptase (b) levels in peritoneal fluid were a significantly higher in the 10 and 12 mmHg intervention groups than the control group (*p* < 0.05), while chymase (c) levels were similar in both groups (*p* > 0.05).

Histamine and tryptase levels in peritoneal fluid were significantly higher in the 10 and 12 mm Hg intervention groups than the control group (histamine: 0.04 ± 0.02 *vs.* 0.03 ± 0.02 *vs.* 0.04 ± 0.035 *vs.* 0.50 ± 0.35 *vs.* 0.41 ± 0.41 ng/mL for control, 5-, 8-, 10-, and 12-mmHg, respectively, *p* < 0.05; and tryptase: 0.48 ± 0.02 *vs.* 0.56 ± 0.07 *vs.* 0.53 ± 0.17 *vs.* 0.69 ± 0.11 *vs.* 0.65 ± 0.05 ng/ml for control, 5-, 8-, 10-, and 12-mmHg, respectively, p < 0.05). Chymase levels were similar among groups (0.96 [range, 0.8–1.19] *vs.* 0.99 [range, 0.66–1.06] *vs.* 0.96 [range, 0.68–1.51] *vs.* 1.04 [range, 1.03–1.10] *vs.* 1.05 [rage, 0.91–1.1] ng/ml, for control, 5-, 8-, 10-, and 12-mmHg, respectively, *p* > 0.05). (Fig. 1a–c).

### 3.2 Extracellular matrix thickness following surgery

**Fig. 2:**
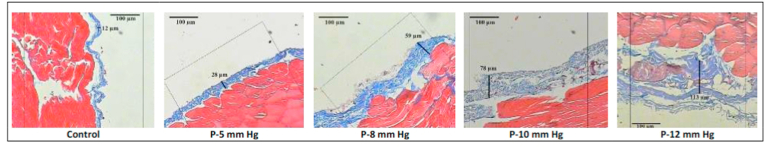
Histopathological findings of extracellular matrix thickness after laparoscopic procedures. The increasing insufflation pressure increases ECM thickness of the parietal peritoneum tissue (Masson trichrome staining, 40x magnifications).

**Fig. 3:**
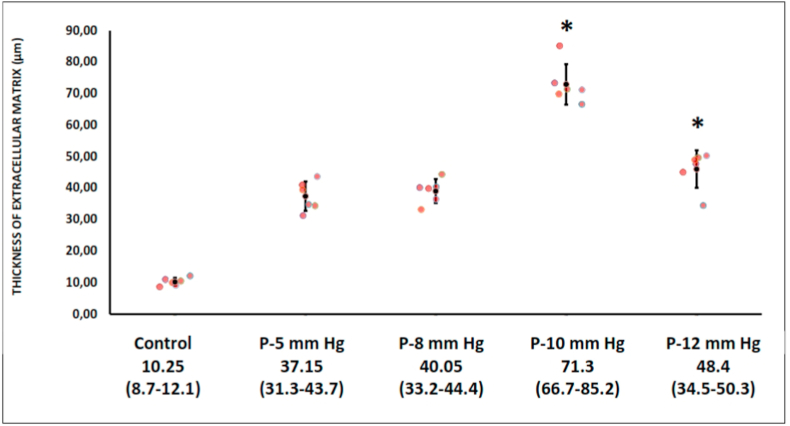
The thickness of the extra-cellular matrix was strongly increased after the laparoscopic procedures compared to the control group (*p* < 0.05).

The ECM was significantly thicker in the intervention groups at 10- and 12-mm Hg than in the control group (10.25 [range, 8.7–12.1] vs. 37.15 [range, 31.3–43.7] vs. 40.05 [range, 33.2–44.4] vs. 71.3 [range, 66.7–85.2] vs. 48.4 [range, 34.5–50.3] μm, for control, 5-, 8-, 10-, and 12-mmHg, respectively, *p* < 0.05 (Fig. 2, Fig. 3).

### 3.3 Intra-abdominal scoring system after procedure

**Fig. 4:**
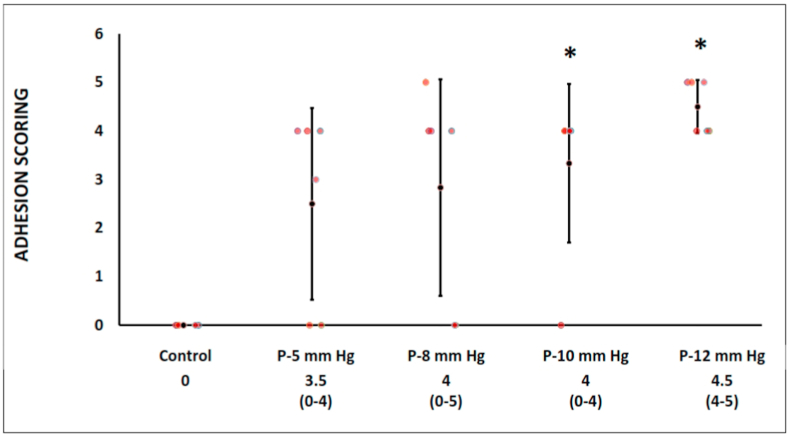
The intra-abdominal scoring was significantly different between laparoscopic and control groups (*p* < 0.05).

The intra-abdominal scoring was significantly higher in the intervention groups at 10- and 12- mm Hg than control group (0 *vs.* 3.5 [range, 0–4] *vs.* 4 [range, 0–5] *vs.* 4 [range, 0–4] *vs*. 4.5 [range, 4–5], for control, 5-, 8-, 10-, and 12-mmHg, respectively, *p* < 0.05 (Fig. 4).

## 4 Discussion

Laparoscopic pneumo-peritoneum causes hypoxia and ischemia-reperfusion injury (especially during desufflation), oxidative stress, and cell damage [20,21]. Cell damage triggers the production of Damage Associated Molecular Patterns (DAMPs) and inflammatory responses [22,23]. Mast cells and other innate immune cells will become active for homeostasis [20]. Mast cells have unique features compared to other inflammatory cells [24], becoming mature in tissue, with longer life, and a role in the fibrosis process [25]. These pathological conditions cause excessive mast cell infiltration and degranulation [[26], [27], [28]]. Our study identified an increase in histamine and tryptase levels in laparoscopic procedure pressures of 10- and 12-mm Hg. The pneumoperitoneum procedure involves non-immunological (physical stimulation) [29] and causes mast cell degranulation [17]. Hypoxia triggers anaerobic respiration, and adenosine triphosphate (ATP) deficiency, which results in interference of the ATPase dependent on mast cell membrane channel. This mechanism disrupts water, ion, and cellular homeostasis [30]. Hypoxia causes the activation of the C3a and C5a molecules and activates the G Protein-Coupled Receptors (GPCR) receptors resulting in degranulation [31]. The pressure and cold of CO_2_ pneumoperitoneum cause interference to the Ca^2+^ channel of mast cells [29]. Lipid, protein, and deoxyribonucleic acid (DNA) peroxidation due to Reactive Oxygen Species (ROS) also cause mast cell degranulation [32]. The mast cell is a non-excitable immunological cell that is sensitive to physical trauma [33,34]. Transient receptor potential canonical (TRPC) Ca^2+^ channel is sensitive to temperature changes. Calcium release-activated channels (CRAC) [35] and transient receptor potential vanilloid (TRPV4) [36] are mechanosensitive (MS) channels that are sensitive to pressure. The voltage-dependent anion channel (VDAC) mitochondria Ca^2+^ channel regulates cytoplasmic levels and causes mast cell degranulation if the VDAC function has interfered [29].

Mast cell degranulation releases histamine and proteases. Mast cell histamine and proteases are high in fibrosis areas [37]. Histamine causes vascular vasodilation, and increases molecular cell adhesion, and modulates the migration and proliferation of fibroblasts [25]. Mast cell tryptase and chymase increase transforming growth factor-beta (TGF-β) activity, decrease the cell tight junction affinity and become a pro-fibrotic protein [25,38]. TGF-β triggers mesothelial-transformation increases the ECM thickness [39] and leads to fibrosis [40]. Tryptase and chymase are the angiogenic factors [25] and trigger ECM thickness. Chymase results in the degradation of the enzymes' vitronectin and fibronectin, transforming the matrix metallopeptidase 9 (pro-MMP9) into active forms and modulate the thickening of ECM [7]. Tryptase causes degradation of type 4 collagen as the main structure of the basement membrane [41]. Tryptase and chymase inhibit the fibrinolysis enzymes (tissue plasminogen activator/tPA and urokinase plasminogen activator/uPA), increasing fibrin [25]. They activate the protease-activated receptor 2 (PAR-2) receptors, causing degradation of the cell junction components, which causes mesothelial release from the basement membrane [42]. Different from research conducted by Berdun et al. [17], our study found no significant increase in chymase levels. It was suspected that the mast cell chymase population is lower than tryptase in mesothelial tissue. This finding is most likely related to the specific trauma of laparoscopy.

Our study found an increase in the ECM and intra-abdominal scoring in laparoscopy over 10 mm Hg. The ECM is a 3-dimensional structure consisting of collagen, enzymes, glycoproteins (proteoglycans), and extracellular vesicles (Deoxyribonucleic acid/DNA, Ribonucleic acid/RNA, and Matrix-bound Nano vesicles/MBVs) [43,44]. The effect of laparoscopy is multi-factorial on the 3-dimensional structure of ECM, including mast cell degranulation [45]. Laparoscopy procedures trigger proliferation, differentiation, migration, and ECM formation towards fibrosis, due to an imbalance of the coagulation and fibrinolysis process [46].

Although good and simple to apply clinically, the intraabdominal scoring system should be done in more studies, particularly in humans. Future research is needed on mast cell stabilizers to prevent intra-abdominal adhesion to further confirm our findings.

## 5 Conclusions

Laparoscopic procedures increase the release of mast cell mediators in peritoneal fluid, the thickness of extracellular matrix and intraabdominal adhesion scoring in rats, implying that it might increase the possibility of intrabdominal adhesion in humans.

## Conflicts of interest

All authors declare that they have no conflict of interest.

## Consent

Not applicable.
