# Supplementary material for: Laparoscopic procedures impact on mast cell mediators, extracellular matrix and adhesion scoring system in rats
Source: Ann Med Surg (Lond). 2020 Sep 2;58:102–6. doi: 10.1016/j.amsu.2020.08.043 (PMC7490447; doi:10.1016/j.amsu.2020.08.043)
Supplement: Multimedia component 2 [file mmc2.docx]

**Table 2.** Animal Research: Reporting In Vivo Experiments: The ARRIVE guidelines.

|  |  |  | |
| --- | --- | --- | --- |
| **Title** | **ITEM** | **RECOMMENDATION** | |
| **TITLE** | 1 | Laparoscopic procedures impact on mast cell mediators, extracellular matrix and adhesion scoring system in rats | |
| **ABSTRACT** | 2 | **Background:** Laparoscopic procedures at specific pressures potentially cause intra-abdominal adhesion. However, the pathomechanism of intra-abdominal adhesion in laparoscopy is still challenging to understand. The release of mast cell mediators due to mast cell degranulation is presumed to play a role in intra-abdominal adhesion. Our objective was to determine the impact of laparoscopic procedures on 1. mast cell mediators’ level, including histamine, tryptase, and chymase; 2. the thickness of the extracellular matrix (ECM) of peritoneal tissue; and 3. intraabdominal adhesion scoring system.  **Materials and methods:** Thirty male Sprague-Dawley rats were grouped into five groups (n = 6 per group): one control group and four intervention groups to which 60 minutes insufflation was performed using carbon dioxide (CO2) at 5, 8, 10 and 12 mmHg, respectively. Seven days after laparoscopy, we euthanized and evaluated the levels of histamine, tryptase, and chymase of peritoneal fluid, the thickness of ECM of peritoneal tissue, and intra-abdominal adhesion scoring system.  **Results:** Histamine and tryptase levels in peritoneal fluid were significantly higher at the 10 and 12 mm Hg intervention than the control group (histamine: 0.04±0.02 vs. 0.03±0.02 vs. 0.04±0.035 vs. 0.50±0.35 vs. 0.41±0.41 ng/mL for control, 5-, 8-, 10-, and 12-mmHg, respectively, p<0.05; and tryptase: 0.48±0.02 vs. 0.56±0.07 vs. 0.53±0.17 vs. 0.69±0.11 vs. 0.65±0.05 ng/ml for control, 5-, 8-, 10-, and 12-mmHg, respectively, p<0.05). The ECM was significantly thicker in the intervention groups at 10- and 12-mm Hg than in the control group (10.25 [range, 8.7-12.1] vs. 37.15 [range, 31.3-43.7] vs. 40.05 [range, 33.2-44.4] vs. 71.3 [range, 66.7-85.2] vs. 48.4 [range, 34.5-50.3] μm, for control, 5-, 8-, 10-, and 12-mmHg, respectively, p<0.05. Moreover, the intra-abdominal scoring was significantly higher in the intervention groups at 10- and 12- mm Hg than control group (0 vs. 3.5 [range, 0-4] vs. 4 [range, 0-5] vs. 4 [range, 0-4] vs. 4.5 [range, 4-5], for control, 5-, 8-, 10-, and 12-mmHg, respectively, p<0.05.  **Conclusions:** Laparoscopic procedures increase the release of mast cell mediators in peritoneal fluid, the thickness of ECM, and intraabdominal adhesion scoring in rats, implying that it might increase the possibility of intrabdominal adhesion in humans. | |
| **INTRODUCTION** |  |  | |
| **Background** | 3 | Laparoscopic procedures at specific pressures potentially cause intra-abdominal adhesion. However, the pathomechanism of intra-abdominal adhesion in laparoscopy is still challenging to understand. The release of mast cell mediators due to mast cell degranulation is presumed to play a role in intra-abdominal adhesion. | |
| **Objectives** | 4 | Our objective was to determine the impact of laparoscopic procedures on 1. mast cell mediators’ level, including histamine, tryptase, and chymase; 2. the thickness of the extracellular matrix (ECM) of peritoneal tissue; and 3. intraabdominal adhesion scoring system. | |
| **METHODS** |  |  | |
| **Ethical statement** | 5 | Our study strictly followed the ethical and euthanasia guidelines for animal research (http://risetcenterfk.ulm.ac.id/euthanasia/). The Animal Experimentation Ethical Committee, Research Center, Faculty of Medicine, Universitas Lambung Mangkurat, Banjarmasin, Indonesia, had approved our research (No.282/KEPK-FK.UNLAM/EC/VII/2019).  This study was conducted according to the 3R5F principles of experimental animal studies | |
| **Study design** | 6 | 1. Thirty male, 200-250 g, and 20-25 weeks old Sprague-Dawley rats (Rattus norvegicus) were randomly computerize divided into a control group and four intervention groups. 2. Sprague-Dawley rats (Rattus norvegicus) were randomly computerize divided into a control group and four intervention groups. 3. The rats were kept in standard breeding-housing (maintained 20 ± 20 C temperature, 12 h light/dark cycle), with health monitor, and 7 days of acclimation | |
| **Experimental**  **procedures** | 7 | 1. According to the previous study (6), sixty-minute laparoscopy was performed in a sterile area after shaving and povidone-iodine application. Pneumoperitoneum used standard CO2 and CO2 automatic-insufflators (Gimmi, Gimmi®GmbH, Germany, 2000).   Health monitor was done each day  Ten mg/kg BW intramuscular injections of ketamine hydrochloride (KTM-10; PT Guardian Pharmatama, No. Reg. DKL0408013443B1) were used for anesthesia.   1. Decapitation was performed to euthanize the rats on the 7th-day after laparoscopy 2. The experiments were conducted in the Chemical/Biochemical Laboratory, the Anatomical Pathology Laboratory, Faculty of Medicine, Universitas Lambung Mangkurat, Banjarmasin, Indonesia. 3. The 5,8, 10, 12 mmHg represent low, medium and high pressures on laparoscopy | |
| **Experimental**  **animals** | 8 | 1. Thirty males (10), 200-250 g, and 20-25 weeks old Sprague-Dawley rats (Rattus norvegicus) were randomly computerize divided into a control group and four intervention groups. 2. According to the previous study, rats’ males prefer because of have higher blood volume dan avoid the estrus phase (6) | |
| **Housing and husbandry** | 9 | 1. The rats were kept in standard breeding-housing 2. The breeding-housing maintained 20 ± 20 C temperature, 12 h light/dark cycle), standard food, mineral water, health monitor, and 7 days of acclimation 3. Health monitor was done each day before and after the operation, and also post-euthanasia care. | |
| **Sample size** | 10 | 1. Thirty males (10) Sprague-Dawley rats (Rattus norvegicus) were randomly computerize divided into a control group and four intervention groups. 2. Federer formula was used for the sample size calculation 3. This study was conducted according to the 3R5F principles of experimental animal studies (8,9). | |
| **Allocating animals to experimental groups** | 11 | 1. Thirty males (10) Sprague-Dawley rats (Rattus norvegicus) were randomly computerize divided into a control group and four intervention groups. 2. The control group (n = 6) did not receive pneumoperitoneum. The intervention groups of P-5 mmHg, P-8 mmHg, P-10 mmHg, and P-12 mmHg (all n = 6) were given 5, 8, 10, and 12 mmHg CO_2_ pneumoperitoneum, respectively. | |
| **Experimental outcomes** | 12 | 1. Our objective was to determine the impact of laparoscopic procedures on 1. mast cell mediators’ level, including histamine, tryptase, and chymase; 2. the thickness of the extracellular matrix (ECM) of peritoneal tissue; and 3. intraabdominal adhesion scoring system. | |
| **Statistical methods** | 13 | 1. Our study results were presented as numbers, percentages, mean ± standard deviation (SD), and median (range, minimum-maximum). Data were analyzed for normality (using Kolmogorov–Smirnov, and Shapiro–Wilk tests), homogeneity using Levine’s test, and underwent data transformation methods (power > 1, inverse, log10, and square root). One-way ANOVA and the post-hoc LSD tests were used for normally and homogeneously distributed data. One-way test of Equality of Means and the post-hoc Games-Howell test were used for normally but non-homogeneously distributed data. Kruskal-Wallis and post-hoc Mann-Whitney tests were used for non-normally distributed data. 2. The histamine and tryptase levels are in ng/mL; the extracellular matrix thickness is in μm; The intra-abdominal scoring is in absolute number. 3. With a confidence interval of 95% (α=0.05), the analysis used IBM SPSS version 23.0 and Microsoft Excel 2010. | |
| **RESULT** |  |  | |
| **Baseline data** | 14 | Thirty males (10), 200-250 g, and 20-25 weeks old Sprague-Dawley rats (Rattus norvegicus) were randomly computerize divided into a control group and four intervention groups. The rats were kept in standard breeding-housing (maintained 20 ± 20 C temperature, 12 h light/dark cycle), standard food, mineral water, health monitor, and 7 days of acclimation) (11). Normal rats were characterized by normal vital signs, active and no eye and anal secretions | |
| **Numbers analyzed** | 15 | 1. Thirty males (10), 200-250 g, and 20-25 weeks old Sprague-Dawley rats (Rattus norvegicus) were randomly computerize divided into a control group and four intervention groups. The control group (n = 6) did not receive pneumoperitoneum. The intervention groups of P-5 mmHg, P-8 mmHg, P-10 mmHg, and P-12 mmHg (all n = 6) were given 5, 8, 10, and 12 mmHg CO2 pneumoperitoneum, respectively (6,12). 2. The sick and dead rats were excluded from the study and replaced with healthy. | |
| **Outcomes and estimation** | 16 | **Mast cell mediators’ level after laparoscopic procedures**  Histamine and tryptase levels in peritoneal fluid were significantly higher in the 10 and 12 mm Hg intervention groups than the control group (histamine: 0.04±0.02 vs. 0.03±0.02 vs. 0.04±0.035 vs. 0.50±0.35 vs. 0.41±0.41 ng/mL for control, 5-, 8-, 10-, and 12-mmHg, respectively, p<0.05; and tryptase: 0.48±0.02 vs. 0.56±0.07 vs. 0.53±0.17 vs. 0.69±0.11 vs. 0.65±0.05 ng/ml for control, 5-, 8-, 10-, and 12-mmHg, respectively, p<0.05). Chymase levels were similar among groups (0.96 [range, 0.8-1.19] vs. 0.99 [range, 0.66-1.06] vs. 0.96 [range, 0.68-1.51] vs. 1.04 [range, 1.03-1.10] vs. 1.05 [rage, 0.91-1.1] ng/ml, for control, 5-, 8-, 10-, and 12-mmHg, respectively, p>0.05).  **Extracellular matrix thickness following surgery**  The ECM was significantly thicker in the intervention groups at 10- and 12-mm Hg than in the control group (10.25 [range, 8.7-12.1] vs. 37.15 [range, 31.3-43.7] vs. 40.05 [range, 33.2-44.4] vs. 71.3 [range, 66.7-85.2] vs. 48.4 [range, 34.5-50.3] μm, for control, 5-, 8-, 10-, and 12-mmHg, respectively, p<0.05  **Intra-abdominal scoring system after procedure**  The intra-abdominal scoring was significantly higher in the intervention groups at 10- and 12- mm Hg than control group (0 vs. 3.5 [range, 0-4] vs. 4 [range, 0-5] vs. 4 [range, 0-4] vs. 4.5 [range, 4-5], for control, 5-, 8-, 10-, and 12-mmHg, respectively, p<0.05 | |
| **Adverse events** | 17 | 1. This study was conducted according to the 3R5F principles of experimental animal studies (including pain). 2. Ten mg/kg BW intramuscular injections of ketamine hydrochloride (KTM-10; PT Guardian Pharmatama, No. Reg. DKL0408013443B1) were used for anesthesia. | |
| **DISCUSSION** |  |  | |
| **Interpretation/scientific implication** | 18 | 1. There were some results support previous studies, and some are different from previous studies. Laparoscopic pneumo-peritoneum causes hypoxia and ischemia-reperfusion injury (especially during desufflation), oxidative stress, and cell damage. Our study identified an increase in histamine and tryptase levels in laparoscopic procedure pressures of 10- and 12-mm Hg. The pneumoperitoneum procedure involves non-immunological (physical stimulation). Mast cell degranulation releases histamine and proteases. Histamine causes vascular vasodilation, and increases molecular cell adhesion, and modulates the migration and proliferation of fibroblasts (25). Mast cell tryptase and chymase increase transforming growth factor-beta (TGF-β) activity, decrease the cell tight junction affinity and become a pro-fibrotic protein. Different from research conducted by Berdun et al. (17), our study found no significant increase in chymase levels. Our study found an increase in the ECM and intra-abdominal scoring in laparoscopy over 10 mm Hg. 2. Although good and simple to apply clinically, the intraabdominal scoring system should be done in more studies, particularly in humans. 3. Based on the Federer formula for the number of rats is valid and representative. | |
| **Generalizability/translation** | 19 | It is hoped that the use of rats as experimental animals can be applied to humans because of their physiological similarities | |
| **Funding** | 20 | Personal funding | |
|  |  |  | |
| ^a^Schulz, et al. (2010) [24]  doi:10.1371/journal.pbio.1000412.t002 | | |  |
